# Strategien des Entlassmanagements in deutschen Allgemeinkrankenhäusern: Deutschlandweite Befragung von Verantwortlichen des klinischen Risikomanagements

**DOI:** 10.1007/s00103-024-03846-0

**Published:** 2024-03-01

**Authors:** Matthias Marsall, Matthias Weigl, Martina Schmiedhofer, Karl Blum, Hannah Rösner, Reinhard Strametz, Nikoloz Gambashidze

**Affiliations:** 1 Institut für Patientensicherheit (IfPS), Universitätsklinikum Bonn, Venusberg-Campus 1, Gebäude A 02, 53127 Bonn, Deutschland; 2 Aktionsbündnis Patientensicherheit e. V., Berlin, Deutschland; 3 Deutsches Krankenhausinstitut, Düsseldorf, Deutschland; 4 Wiesbaden Business School, Rhein Main University of Applied Sciences, Wiesbaden, Deutschland

**Keywords:** Patientensicherheit, Qualitätsmanagement, Klinisches Risikomanagement, Sektorenübergreifende Übergänge, Transitionsprozesse, Patient safety, Quality management, Clinical risk management, Care transition, Transition processes

## Abstract

**Hintergrund:**

Sektorenübergreifende Übergänge aus der stationären Versorgung sind mit Risiken für die Patientensicherheit verbunden. Im Jahr 2017 wurde der Rahmenvertrag über das Entlassmanagement rechtlich verankert. Zur Umsetzung von Maßnahmen zur Gewährleistung sicherer Übergänge von Patient:innen nach stationärer Behandlung fehlen in Deutschland bisher empirische Daten. Ziel dieser Studie ist ein Überblick über die von deutschen Allgemeinkrankenhäusern umgesetzten Strategien des Entlassmanagements.

**Methode:**

Zwischen März und Mai 2022 wurden in einer deutschlandweiten Befragung konkrete Strategien des Entlassmanagements sowie strukturelle und organisationsbezogene Merkmale von 401 Allgemeinkrankenhäusern erfasst und im Anschluss deskriptive Statistiken und Gruppenvergleiche durchgeführt.

**Ergebnisse:**

7 von 9 erfragten Strategien wurden in > 95 % der Häuser umgesetzt. Die Evaluation der Entlassungsplanung wurde nur in 61 % der Häuser umgesetzt, die systematische Dokumentation, Analyse und Evaluation der Wiederaufnahme in 54 %. Häuser mit einer höheren Anzahl Planbetten berichteten signifikant seltener über „frühzeitige Kontaktaufnahme mit Nachversorgenden“ und „Organisation des nahtlosen Übergangs in die Anschlussversorgung“.

**Diskussion:**

Ein Großteil der Strategien im Entlassmanagement aus der stationären Behandlung wird in deutschen Allgemeinkrankenhäusern umgesetzt. Allerdings werden Maßnahmen zur Evaluation und systematischen Analyse von Entlassungsprozessen sowie Wiederaufnahmen von Patient:innen nur teilweise umgesetzt. Diese sind jedoch notwendig, um Entlassungsprozesse und Verbesserungspotenziale systematisch zu bewerten.

**Zusatzmaterial online:**

Zusätzliche Informationen sind in der Online-Version dieses Artikels (10.1007/s00103-024-03846-0) enthalten.

## Abstract (en)

**Background:**

Transitions from inpatient care are associated with risks for the safety of patients. In 2017, the framework agreement on discharge management was legally defined. There is currently a lack of empirical data in Germany on the implementation of measures to ensure safe transitions of patients after inpatient care. The aim of this study is to provide an overview of the discharge management strategies implemented by German general hospitals.

**Methods:**

Between March and May 2022, specific discharge management strategies as well as structural and organizational characteristics were assessed in a nationwide survey of 401 general hospitals, and descriptive statistics and group comparisons were performed.

**Results:**

Seven of nine strategies surveyed were implemented in > 95% of all hospitals. The evaluation of discharge planning was only implemented in 61% of the hospitals, and systematic documentation, analysis, and evaluation of readmissions in 54%. Hospitals with a higher number of hospital beds reported significantly less often about “early contact with follow-up care providers” and “organization of a seamless transition to follow-up care.”

**Discussion:**

A large part of the strategies in discharge management from inpatient treatment is implemented in German general hospitals. However, measures for evaluation and the systematic analysis of discharge processes and readmissions of patients have only been partially implemented. However, these are necessary to systematically evaluate and potentially improve the discharge processes.

## Hintergrund

Die Organisation sicherer Übergänge von Patient:innen von der stationären Behandlung im Krankenhaus in die ambulante häusliche Versorgung ist eine der zentralen Säulen der Patientensicherheit [1]. International gibt es umfassende Evidenz, dass Mängel in der Gestaltung der Entlassungsprozesse mit negativen Ergebnissen wie ungeplanten Wiedereinweisungen [2], Medikationskomplikationen [3, 4] und geringerer selbstberichteter Gesundheit der Patient:innen [5–7] assoziiert sind.

In der Konsequenz wurde zur Stärkung der Patientensicherheit in der transsektoralen Versorgung das Entlassmanagement in Deutschland rechtlich verankert (Rahmenvertrag über ein Entlassmanagement beim Übergang in die Versorgung nach Krankenhausbehandlung)^1^. Der Gesetzgeber hat konkrete Vorgehensweisen definiert, die im Entlassmanagement zu gewährleisten sind. Das Deutsche Netzwerk für Qualitätsentwicklung in der Pflege hat zudem Struktur‑, Prozess- und Ergebniskriterien als Expertenstandards für das Entlassmanagement in der Pflege definiert [8]. Gleichwohl bedarf die Umsetzung der Strategien im klinischen Alltag zusätzlicher Ressourcen und ist mit erheblichen (bürokratischen) Herausforderungen verbunden [9, 10].

Trotz der verbindlichen Festlegung des Entlassmanagements als Teil der stationären Behandlung im Jahr 2017 gibt es zum konkreten Umsetzungsstand dieser Strategien keine systematische empirische Übersicht oder Bestandsaufnahme. Daher war das Ziel dieser Studie, erstmalig einen empirischen Überblick über den Umsetzungsstand des Entlassmanagements in Allgemeinkrankenhäusern in Deutschland zu erhalten. Mit der Befragung von Verantwortlichen für das klinische Risikomanagement (kRM) war die detaillierte Analyse des aktuellen Umsetzungsstandes konkreter Strategien des Entlassmanagements in deutschen Allgemeinkrankenhäusern sowie die Stratifizierung der Ergebnisse über verschiedene Struktur- und Organisationsmerkmale (SOM) möglich.

Erstes Untersuchungsziel war die Erfassung des Umsetzungsstands von 9 vorab definierten Strategien und Praktiken im Entlassmanagement, um einen Überblick zum Stand der Qualitätssicherung der transsektoralen Versorgung in deutschen Allgemeinkrankenhäusern zu erhalten. Das zweite Untersuchungsziel war die Prüfung, ob die Umsetzungsstände der verschiedenen Maßnahmen durch SOM der Krankenhäuser determiniert sind.

## Methode

### Studiendesign und Setting

Diese Studie war Teil der deutschlandweiten Befragung zum Umsetzungsstand des kRM (Projekt KHaSiMiR 21)^2^. Die Daten wurden mittels einer Online-Querschnittsbefragung (Unipark, Tivian GmbH) durch das Deutsche Krankenhausinstitut (DKI) erhoben. Zusätzlich wurden SOM aus einer Datenbank des DKI ausgeleitet. In der Befragung wurden verschiedene Strategien zur Stärkung der Patientensicherheit, einschließlich konkreter Strategien des Entlassmanagements, erfasst. Die Zusammenführung der Daten aus der Onlinebefragung mit den aus der DKI-Datenbank ausgeleiteten SOM erfolgte mittels eines individuellen Codes. Die Auswertung wurde mit ausschließlich anonymisierten Daten im Institut für Patientensicherheit durchgeführt. Für die freiwillige Teilnahme an der Onlinestudie ohne Erfassung personenbezogener Daten wurde kein Votum der zuständigen Ethikkommission eingeholt. Die Studienergebnisse werden unter Berücksichtigung der „Strengthening the Reporting of Observational Studies in Epidemiology“ (STROBE) Guidelines [11] berichtet.

### Stichprobe

Zwischen März und Mai 2022 wurden die Geschäftsführungen von Allgemeinkrankenhäusern mit 50 und mehr Krankenhausbetten in Deutschland mit der Bitte um die Beantwortung des Fragebogens kontaktiert. Die Gesamtheit der eingeschlossenen Kliniken wurde definiert als gemäß § 108 Fünftes Sozialgesetzbuch (SGB V) zugelassene Allgemeinkrankenhäuser ab 50 Betten. Die betreffenden Allgemeinkrankenhäuser (*N* = 1411) wurden aus der DKI-Datenbank ausgeleitet. 401 Personen nahmen an der Befragung teil (Rücklaufquote: 28,4 %). Davon gaben 113 Personen an, den Fragebogen für mehr als eine Klinik (z. B. Klinikverbünde) ausgefüllt zu haben. Nach einer Datenbereinigung aufgrund unklarer Angaben zur Anzahl von im kRM beschäftigten Personen wurden 2 Fragebögen ausgeschlossen. Weitere 4 Fragebögen wurden ausgeschlossen, weil eine Bettenanzahl unter 50 bzw. eine sehr hohe Bettenanzahl angegeben wurde. Die für die Analyse verwendete Stichprobe umfasste somit 395 Datensätze.

### Instrumente

#### Strategien zum Entlassmanagement

Die 9 erfassten Entlassmanagementstrategien wurden aus dem zuvor vom DKI im „Krankenhaus Barometer 2018“^3^ verwendeten Fragebogen abgeleitet und mithilfe des von der Agency for Healthcare Research and Quality (AHRQ) entwickelten Re-Engineered-Discharge-Programms erweitert [12, 13]. In Tabelle Z1 im Onlinematerial finden sich die Fragen, die für das deutsche Versorgungssystem adaptiert und mit dem Rahmenvertrag über ein Entlassmanagement^4^ sowie nach Expertenstandards des Deutschen Netzwerks für Qualitätsentwicklung in der Pflege harmonisiert wurden [8]. Der Umsetzungsstand wurde mittels einer Skala von 1 = „Nein und nicht geplant“ bis 4 = „Vollständig oder weitgehend umgesetzt“ erfasst.

#### Strukturelle Merkmale der befragten Krankenhäuser

Über den Fragebogen wurde erfasst, wie viele Personen im kRM beschäftigt sind und wie das kRM strukturell organisiert ist (zentral, dezentral, beides, durch externen Dienstleister). Darüber hinaus wurde die Anzahl der Planbetten erhoben. Sofern diese Angabe im Fragebogen fehlte, wurde sie durch den Eintrag der DKI-Datenbank ergänzt. Aus der Angabe wurde die Bettengrößenklasse abgeleitet (50–299 Betten, 300–599 Betten, ≥ 600 Betten). Aus der DKI-Datenbank wurden die Trägerart (öffentlich, privat, freigemeinnützig) und die Krankenhausart (Universitätsklinik, Plankrankenhaus, Haus mit Versorgungsvertrag) sowie das Bundesland übernommen.

### Analysen

Im ersten Schritt wurden die SOM der Allgemeinkrankenhäuser deskriptiv-statistisch durch absolute Zahlen und Prozente (für kategoriale Variablen) dargestellt. Deskriptive Statistiken für kontinuierliche Variablen wurden aufgrund der hohen Antwortvarianz durch Median und Interquartilsabstand (IQR) angegeben. Im zweiten Schritt wurden die Antworten für die 9 Strategien zum Umsetzungsstand des Entlassmanagements deskriptiv ausgewertet. Anschließend wurden die Ergebnisse für die SOM der Allgemeinkrankenhäuser stratifiziert, um potenzielle Unterschiede zu untersuchen. Diese wurden inferenzstatistisch durch Varianzanalysen (ANOVA) und Regressionsanalysen untersucht. Dabei wurde das Signifikanzniveau von 5 % für multiples Testen adjustiert (für jedes Item, Bonferroni-korrigiert [14]). Da für jedes Item 6 Tests (entsprechend den 6 SOM) durchgeführt wurden, wurde das Signifikanzniveau auf α = 0,05/6 = 0,008 festgelegt. Alle Analysen wurden mit R und RStudio durchgeführt [15, 16].

## Ergebnisse

### Strukturelle Merkmale der befragten Allgemeinkrankenhäuser

Die Mehrheit der teilnehmenden Allgemeinkrankenhäuser waren Plankrankenhäuser in öffentlicher oder freigemeinnütziger Trägerschaft. Die Anzahl der Planbetten variierte von 50–2537 Betten, mit einem Median von 373 Betten und einem IQR von 421 Betten. Der Großteil der Häuser war in Bettengrößenklassen von unter 600 Betten. Für das kRM war am häufigsten eine Person verantwortlich (Median = 2, IQR = 2). Die Bundesländer Nordrhein-Westfalen und Bayern machten zusammen 43,8 % der Rückmeldungen aus. Eine Übersicht über die SOM der untersuchten Häuser sowie die jeweiligen Proportionen in Bezug auf die Grundgesamtheit der angeschriebenen Allgemeinkrankenhäuser findet sich in Tab. 1.

**Table 1:** 

	Stichprobe		Grundgesamtheit		Rücklauf
Merkmale	*N*	%	*N*	%	%
*Trägerart*					
Öffentlich	162	41,0	472	33,5	34,3
Freigemeinnützig	182	46,1	568	40,3	32,0
Privat	44	11,1	339	24,0	13,0
Keine Angabe	7	1,8	32 (unbekannt)^3^	2,3	–
*Bettengrößenklasse*					
50–299 Betten	159	40,3	922	65,3	17,2
300–599 Betten	131	33,2	350	24,8	37,4
Ab 600 Betten	105	26,6	139	9,9	75,5
*Krankenhausart*					
Universitätsklinik	18	4,6	38	2,7	47,4
Plankrankenhaus	368	93,2	1326	94,0	27,8
Krankenhaus mit Versorgungsvertrag	6	1,5	43	3,0	14,0
Keine Angabe	3	0,8	4 (unbekannt)^3^	0,3	–
*Anzahl Personen im kRM*^*1,2*^					
Keine	3	0,8	–	–	–
1 Person	125	31,6	–	–	–
2 Personen	110	27,8	–	–	–
3 Personen	53	13,4	–	–	–
4 Personen	18	4,6	–	–	–
5–50 Personen	41	10,4	–	–	–
Keine Angabe	45	11,4	–	–	–
*Organisation des kRM*^*2*^					
Zentral	240	60,8	–	–	–
Dezentral	15	3,8	–	–	–
Beides	117	29,6	–	–	–
Durch externe Dienstleister organisiert	1	0,3	–	–	–
Keine Angabe	22	5,6	–	–	–
*Bundesland*					
Baden-Württemberg	30	7,6	153	10,8	19,6
Bayern	81	20,5	231	16,4	35,1
Berlin	13	3,3	46	3,3	28,3
Brandenburg	13	3,3	51	3,6	25,5
Bremen	4	1,0	12	0,9	33,3
Hamburg	9	2,3	27	1,9	33,3
Hessen	33	8,4	105	7,4	31,4
Mecklenburg-Vorpommern	2	0,5	30	2,1	6,7
Niedersachsen	38	9,6	128	9,1	29,7
Nordrhein-Westfalen	92	23,3	326	23,1	28,2
Rheinland-Pfalz	27	6,8	78	5,5	34,6
Saarland	5	1,3	20	1,4	25,0
Sachsen	19	4,8	71	5,0	26,8
Sachsen-Anhalt	10	2,5	45	3,2	22,2
Schleswig-Holstein	11	2,8	43	3,0	25,6
Thüringen	8	2,0	45	3,2	17,8

Stichprobe: *N* = 395; angeschriebene Grundgesamtheit: *N* = 1411

*kRM* klinisches Risikomanagement

^1^Die Angaben von 5–50 Personen wurden zusammengefasst

^2^Keine Angaben zur Grundgesamtheit aus der Datenbank des Deutschen Krankenhausinstituts (DKI) abgeleitet

^3^Die aus der DKI-Datenbank ausgeleiteten Daten waren keiner Trägerart bzw. Krankenhausart zugeordnet

### Umsetzungsstand von Strategien des Entlassmanagements

**Figure 1:**
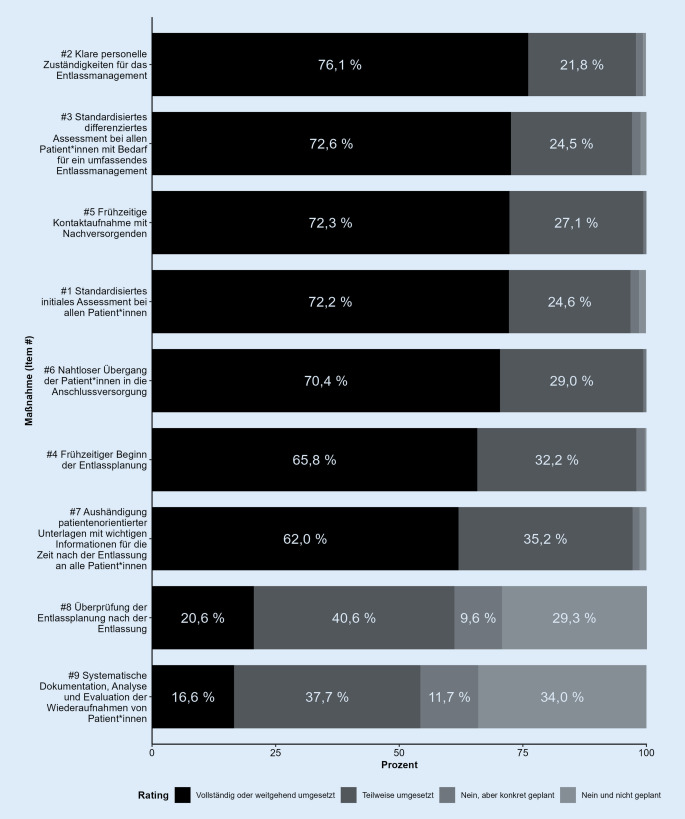

Bei 7 der 9 erfassten Strategien zum Entlassmanagement wurde von mehr als 95 % der Befragten angegeben, dass diese bereits teilweise bis vollständig umgesetzt werden. Demgegenüber wurde bei 39 % der befragten Allgemeinkrankenhäuser keine Evaluation der Entlassungsplanung nach der Entlassung durchgeführt. Ebenso wurden die systematische Dokumentation, Analyse und Evaluation von Wiederaufnahmen in 46 % der befragten Häuser noch nicht durchgeführt. Abb. 1 zeigt die Antwortverteilung der einzelnen Maßnahmen. Eine Übersicht über die deskriptiven Statistiken der 9 Items findet sich in Tabelle Z2 im Onlinematerial.

### Unterschiede des Umsetzungsstandes von Strategien des Entlassmanagements

Zur Überprüfung, ob die Umsetzung einzelner Strategien des Entlassmanagements von SOM der Allgemeinkrankenhäuser determiniert wurde, erfolgte die Stratifizierung nach Trägerart, Bettengrößenklasse, Krankenhausart, Anzahl der im kRM Beschäftigten, Organisationsart des kRM und Bundesland. Für diese Bewertung wurden die positiven Umsetzungsstände (3 = „Teilweise umgesetzt“ und 4 = „Vollständig oder weitgehend umgesetzt“) zusammengefasst. Dies bestätigte, dass die Evaluation der Entlassungsplanung sowie die systematische Dokumentation, Analyse und Evaluation von Wiederaufnahmen seltener umgesetzt wurden als die anderen Strategien des Entlassmanagements (Einzelergebnisse siehe Tabelle Z3 im Onlinematerial).

Inferenzstatistisch wurde gezeigt, dass eine höhere Anzahl der Planbetten mit einem geringeren Umsetzungsstand bei der „frühzeitigen Kontaktaufnahme mit Nachversorgenden“ (Regressionskoeffizient B = −1,76, *p* = 0,003) und der „Organisation des nahtlosen Übergangs in die Anschlussversorgung“ (B = −2,41, *p* < 0,001) assoziiert war. Andere Strategien des Entlassmanagements waren nicht durch die Anzahl der Planbetten determiniert. Für diese Analysen wurde nicht die Bettengrößenklasse, sondern die konkrete Anzahl der Planbetten herangezogen. Alle weiteren untersuchten Zusammenhänge zwischen den SOM und dem Umsetzungsstand der Strategien des Entlassmanagements waren nicht signifikant.

## Diskussion

Patientensicherheit muss nicht nur im Rahmen der Heilbehandlung, sondern auch in der Organisation sicherer Übergänge zwischen verschiedenen Sektoren gewährleistet sein. Insbesondere der Übergang in die selbstorganisierte heimische Versorgung ist für Patient:innen mit erheblichen Risiken verbunden [17]. Effektive Strategien des Entlassmanagements sind Grundvoraussetzung für Patientensicherheit und Qualität in der poststationären Versorgung sowie für die Vermeidung von Wiedereinweisungen. Mit dieser deutschlandweiten Studie wurde untersucht, welche konkreten Strategien des Entlassmanagements in deutschen Allgemeinkrankenhäusern umgesetzt werden.

Mit Bezug auf das erste Untersuchungsziel zeigen die Ergebnisse, dass die meisten der abgefragten Strategien des Entlassmanagements größtenteils umgesetzt sind. Über 95 % der Befragten gaben an, dass klare personelle Zuständigkeiten, ein standardisiertes differenziertes Assessment, eine frühzeitige Kontaktaufnahme mit den Nachsorgenden, ein nahtloser Übergang in die Anschlussversorgung, die frühzeitige Entlassungsplanung sowie die Aushändigung patientenorientierter Unterlagen bereits umgesetzt werden. Mit diesen Strategien werden die Maßnahmen des § 3 des Rahmenvertrags über ein Entlassmanagement beim Übergang in die Versorgung nach Krankenhausbehandlung berücksichtigt.^5^

Dennoch wurden auch kritische Handlungsbedarfe bzw. 2 konkrete Strategien identifiziert, die in den befragten Krankenhäusern bisher nur teilweise oder gar nicht umgesetzt werden: die Evaluation der Entlassungsplanung nach Abschluss der stationären Behandlung sowie die systematische Evaluation der Wiederaufnahmen. Hervorzuheben ist, dass 29 % der Krankenhäuser auch in Zukunft nicht planen, eine Evaluation der Entlassungsplanung nach dem Krankenhausaufenthalt umzusetzen. Ebenso verneinten 34 % der Befragten, zukünftig die Wiedereinweisungen von Patient:innen systematisch auszuwerten. Ungeplante Wiedereinweisungen sind mit hohen Kosten für das Gesundheitssystem verbunden. Schätzungen aus den USA zeigen, dass etwa 30 % der Gesamtausgaben des Gesundheitssystems mit Wiedereinweisungen verbunden sind [18]. Erhöhte Raten von Wiedereinweisungen werden z. B. in den USA durch das Hospital Readmissions Reduction Program bereits durch finanzielle Einbußen bzw. Nachteile für die betreffenden Kliniken geahndet [19]. Gleichzeitig ist eine systematische Aufbereitung ungeplanter Wiedereinweisungen Grundlage für Prozessverbesserungen [13]. Neben finanziellen und organisationsbezogenen Aspekten sind ungeplante Wiedereinweisungen für betroffene Patient:innen mit erheblichem Leid verbunden. Damit zeigen die Ergebnisse dieser Studie explizite Optimierungspotenziale in den Bereichen der systematischen Evaluation von Entlassungsprozessen und Wiedereinweisungen von Patient:innen auf.

Das zweite Untersuchungsziel war es, den Zusammenhang zwischen der Umsetzung einzelner Strategien zum Entlassmanagement und den SOM der Häuser zu identifizieren. Die Analyse zeigte, dass 2 einzelne Strategien, die frühzeitige Kontaktaufnahme mit Nachversorgenden und die Organisation des nahtlosen Übergangs in die Anschlussversorgung, umso seltener umgesetzt werden, je größer die Planbettenzahl eines Hauses ist. Eine mögliche Erklärung könnte sein, dass kleinere Krankenhäuser engere Kontakte zu nachfolgenden Gesundheitsdienstleistern haben und daher der Aufwand zum reibungslosen Übergang der Patient:innen geringer ist. Ein positiver Zusammenhang zwischen der Krankenhausgröße und der Komplikationsrate nach der Entlassung [20] sowie erneuten Wiedereinweisungen [21] wurde bereits belegt. Obwohl dies zumindest teilweise durch einen komplexeren Case-Mix in größeren Krankenhäusern erklärt werden kann [22], können Komplikationen nach der Entlassung und Wiedereinweisungen auch mit der Qualität des Entlassungsprozesses assoziiert sein [23].

Zusammenfassend bietet diese Studie einen systematischen Überblick über den Umsetzungsstand konkreter Strategien des Entlassmanagements in deutschen Allgemeinkrankenhäusern. Dies ist eine wichtige Bestandsaufnahme, um die Patientensicherheit in der transsektoralen Versorgung bei der Entlassung in die häusliche (Selbst‑)Versorgung zu bewerten. Unsere Stichprobe umfasste deutschlandweit Allgemeinkrankenhäuser mit verschiedenen SOM und untermauert eine hohe Validität und Generalisierbarkeit der Studienergebnisse. Mit der Befragung von für das kRM Beauftragten erhielten wir Auskünfte von Personen, die direkt mit den Versorgungsstrukturen des Entlassmanagements befasst sind. Dadurch erhielten wir eine aussagekräftige und zuverlässige Bestandsaufnahme zur Umsetzung von Strategien des Entlassmanagements in Deutschland.

### Limitationen

Wir verwendeten Fragen, die teilweise auf einem vom DKI verwendeten Fragebogen basieren, der an Aspekte des amerikanischen Gesundheitssystems angepasst wurde. Um die Vergleichbarkeit von Ergebnissen verschiedener Studien zu gewährleisten, ist ein validiertes Instrument zur Erfassung des Umsetzungsstandes des Entlassmanagements notwendig. Die Ergebnisse der Studie beschreiben die Situation deutscher Allgemeinkrankenhäuser und sind nicht generalisierbar für andere Einrichtungen, wie Rehabilitationseinrichtungen und Fachkliniken. Durch die Freiwilligkeit der Studienteilnahme und der damit einhergehenden unvollständigen Rücklaufquote ist ein Selektionsbias der Stichprobe nicht auszuschließen. Ferner besteht die Möglichkeit, dass die Studienteilnehmenden von einer nicht anonymen Auswertung der Daten ausgingen, wodurch eine Verzerrung der Antworttendenzen nicht auszuschließen ist. Durch die Erhebung von Selbstberichten können zudem positive Überschätzungen nicht ausgeschlossen werden. Die in dieser Studie berichteten Ergebnisse stellen die Perspektive der für das kRM verantwortlichen Personen in Allgemeinkrankenhäusern dar, ohne andere am Entlassungsprozess beteiligte Akteur:innen, wie z. B. für das Entlassmanagement direkt verantwortliche Personen, Patient:innen und deren Angehörige oder ambulante Versorgungsstrukturen, zu berücksichtigen. Hierdurch zeigen die Ergebnisse einen unvollständigen Ausschnitt über den Stand des Entlassmanagement in deutschen Allgemeinkrankenhäusern. Die in Tab. 1 dargestellten Rücklaufquoten divergieren teilweise in Bezug auf die erfassten SOM, wodurch von einer nicht vollständig repräsentativen Stichprobe und Einschränkungen der Generalisierbarkeit der Studienergebnisse ausgegangen werden muss.

### Implikationen für Forschung und die Versorgungspraxis

Unsere Studie zeigt erstmalig einen Überblick über die Versorgungsstruktur des Entlassmanagements in deutschen Allgemeinkrankenhäusern. Auf unseren Erkenntnissen aufbauende Studien sollten weitere Methoden und Perspektiven, wie z. B. behandlungsbezogene Daten und Outcomes aus Patientenakten, berücksichtigen. Im Sinne der patientenzentrierten Versorgung sollten Patient:innen einbezogen und die Untersuchung von Zusammenhängen zwischen der Umsetzung von Strategien des Entlassmanagements und patientenberichteten Outcomes multiperspektivisch erfolgen [24, 25]. Darüber hinaus sollten auch weitere Schnittstellen der transsektoralen Versorgung, z. B. der Übergang von Patient:innen aus der ambulanten in die stationäre Versorgung, systematisch untersucht werden. Für die Versorgungspraxis bedeuten unsere Ergebnisse, dass viele Strategien des Entlassmanagements bereits umgesetzt werden. Jedoch bieten sich mit der Überprüfung der Entlassungsplanung sowie der systematischen Dokumentation, Analyse und Evaluation der Wiederaufnahmen Potenziale zur Verminderung von Leid für die Patient:innen durch ungeplante Wiedereinweisungen. Darüber hinaus sind diese Strategien bedeutsam für die Verbesserung der Patientensicherheit in der transsektoralen Versorgung und die Kostenreduktion im Gesundheitswesen [18, 26, 27].

## Fazit

Ein großer Teil konkreter Strategien des Entlassmanagements wird in deutschen Allgemeinkrankenhäusern umgesetzt. Die Umsetzung ist dabei außer durch die Anzahl der Planbetten nicht durch weitere strukturelle oder organisationsbezogene Merkmale der Krankenhäuser determiniert. Es zeigen sich Optimierungspotenziale bei der Etablierung der systematischen Evaluation der Entlassungsplanung nach der Entlassung sowie der systematischen Dokumentation, Analyse und Evaluation der Wiederaufnahmen von Patient:innen. Die Analyse und die summative Evaluation von Entlassungsprozessen und deren Outcomes (z. B. Wiedereinweisungen) sind wichtige Strategien zur Stärkung der Patientensicherheit in der transsektoralen Versorgung. Weitere Forschung, z. B. im Rahmen vollständig anonymer Klinikbefragungen von für das Entlassmanagement verantwortlichen Personen sowie unter Einbezug von Patient:innen und ambulanten Versorgungsstrukturen, ist zur umfassenden Beurteilung des Entlassmanagements in deutschen Allgemeinkrankenhäusern notwendig.

### Supplementary Information





## Footnotes

1 Rahmenvertrag Entlassmanagement: https://www.dkgev.de/fileadmin/default/Mediapool/2_Themen/2.3_Versorgung-Struktur/2.3.3_Entlassmanagement/Rahmenvertrag_Entlassmanagement_neu.pdf (Zugriff am 09.12.2023); 10. Änderungsvereinbarung Rahmenvertrag Entlassmanagement vom 22.05.2023: https://www.dkgev.de/fileadmin/default/Mediapool/2_Themen/2.3_Versorgung-Struktur/2.3.3_Entlassmanagement/10._AEnderungsvereinbarung_Rahmenvertrag_Entlassmanagement_22.05.2023.pdf (Zugriff am 09.12.2023).

2 KHaSiMiR 21 – Krankenhausstudie zur Sicherheit durch Management innerklinischer Risiken 2021–22. https://www.aps-ev.de/wp-content/uploads/2023/01/KHaSiMiR_Abschlussbericht_Teil-I.pdf. (Zugriff am 06.03.2023).

3 DKI Krankenhaus Barometer: Umfrage 2018. Deutsches Krankenhausinstitut e. V. https://www.dki.de/fileadmin/forschungsberichte/2018_11_kh_barometer_final.pdf (Zugriff am 16.06.2023).

4 Rahmenvertrag Entlassmanagement: https://www.dkgev.de/fileadmin/default/Mediapool/2_Themen/2.3_Versorgung-Struktur/2.3.3_Entlassmanagement/Rahmenvertrag_Entlassmanagement_neu.pdf (Zugriff am 09.12.2023); 10. Änderungsvereinbarung Rahmenvertrag Entlassmanagement vom 22.05.2023: https://www.dkgev.de/fileadmin/default/Mediapool/2_Themen/2.3_Versorgung-Struktur/2.3.3_Entlassmanagement/10._AEnderungsvereinbarung_Rahmenvertrag_Entlassmanagement_22.05.2023.pdf (Zugriff am 09.12.2023).

5 Rahmenvertrag Entlassmanagement: https://www.dkgev.de/fileadmin/default/Mediapool/2_Themen/2.3_Versorgung-Struktur/2.3.3_Entlassmanagement/Rahmenvertrag_Entlassmanagement_neu.pdf (Zugriff am 09.12.2023); 10. Änderungsvereinbarung Rahmenvertrag Entlassmanagement vom 22.05.2023: https://www.dkgev.de/fileadmin/default/Mediapool/2_Themen/2.3_Versorgung-Struktur/2.3.3_Entlassmanagement/10._AEnderungsvereinbarung_Rahmenvertrag_Entlassmanagement_22.05.2023.pdf (Zugriff am 09.12.2023).
