# Supplementary material for: Discharge management strategies in German general hospitals: A nationwide survey of professionals responsible for clinical risk management
Source: Bundesgesundheitsblatt Gesundheitsforschung Gesundheitsschutz. 2024 Mar 1;67(5):587–94. [Article in German] doi: 10.1007/s00103-024-03846-0 (PMC11093802; doi:10.1007/s00103-024-03846-0)
Supplement: Supplementary file 1 [file 103_2024_3846_MOESM1_ESM.docx]

**Strategien des Entlassmanagements in deutschen Allgemeinkrankenhäusern.**

**Deutschlandweite Befragung von Verantwortlichen des klinischen Risikomanagements**

# Zusätzliches Onlinematerial

## Tabelle Z1: Items zur Erfassung des Umsetzungsstandes des Entlassmanagements

**Fragetext**:

Inwieweit kommen die folgenden Instrumente/Methoden des Entlassmanagements in Ihrer Einrichtung zum Einsatz?

| **Item #** | **Item** |
| --- | --- |
| 1 | Standardisiertes initiales Assessment bei allen Patient:innen |
| 2 | Klare personelle Zuständigkeiten für das Entlassmanagement |
| 3 | Standardisiertes differenziertes Assessment bei allen Patient:innen mit Bedarf für ein umfassendes Entlassmanagement |
| 4 | Frühzeitiger Beginn der Entlassungsplanung |
| 5 | Frühzeitige Kontaktaufnahme mit Nachversorgenden |
| 6 | Nahtloser Übergang der Patient:innen in die Anschlussversorgung |
| 7 | Aushändigung patientenorientierter Unterlagen mit wichtigen Informationen für die Zeit nach der Entlassung an alle Patient:innen |
| 8 | Überprüfung der Entlassungsplanung nach der Entlassung |
| 9 | Systematische Dokumentation, Analyse und Evaluation der Wiederaufnahmen von Patient:innen |

**Antwortformat**:

1 = „Nein und nicht geplant“

2 = „Nein, aber konkret geplant“

3 = „Teilweise umgesetzt“

4 = „Vollständig oder weitgehend umgesetzt“

**Quelle**: Gambashidze, Nikoloz, Karl Blum, Hannah Rösner, Martina Schmiedhofer, Reinhard Strametz, und Matthias Weigl. „KHaSiMiR 21 – Krankenhausstudie zur Sicherheit durch Management innerklinischer Risiken 2021-22“. Krankenhausstudie zur Sicherheit durch Management innerklinischer Risiken: Befragung zum Umsetzungsstand des klinischen Risikomanagements (kRM) in Krankenhäusern und Rehabilitationskliniken in Deutschland 2021 (KHaSiMiR 21).

## Tabelle Z2: Deskriptive Statistiken der Items zum Stand der Umsetzung von Strategien des Entlassmanagements

| **Item #** | **Strategie** | **M** | **SD** | **Missing (%)** | **Umsetzungsstand (%)** | | | |
| --- | --- | --- | --- | --- | --- | --- | --- | --- |
|  |  |  |  |  | **1** | **2** | **3** | **4** |
| 1 | Standardisiertes initiales Assessment | 3,68 | 0,58 | 12,7 | 1,3 | 1,5 | 21,5 | 63,0 |
| 2 | Klare personelle Zuständigkeiten Entlassmanagement | 3,74 | 0,51 | 11,9 | 0,5 | 1,3 | 19,2 | 67,1 |
| 3 | Standardisiertes differenziertes Assessment für Entlassmanagement | 3,69 | 0,57 | 13,2 | 1,0 | 1,5 | 21,3 | 63,0 |
| 4 | Frühzeitiger Beginn Entlassungsplanung | 3,64 | 0,53 | 11,9 | 0,3 | 1,5 | 28,4 | 58,0 |
| 5 | Frühzeitige Kontaktaufnahme mit Nachversorgenden | 3,72 | 0,46 | 12,2 | 0,0 | 0,5 | 23,8 | 63,5 |
| 6 | Nahtloser Übergang in die Anschlussversorgung | 3,70 | 0,47 | 11,9 | 0,0 | 0,5 | 25,6 | 62,0 |
| 7 | Aushändigung patientenorientierter Unterlagen | 3,58 | 0,60 | 12,2 | 1,3 | 1,3 | 30,9 | 54,4 |
| 8 | Evaluation der Entlassungsplanung nach Entlassung | 2,53 | 1,12 | 15,2 | 24,8 | 8,1 | 34,4 | 17,5 |
| 9 | Systematische Evaluation der Wiederaufnahmen | 2,37 | 1,12 | 17,5 | 28,1 | 9,6 | 31,1 | 13,7 |

N = 395. M = Mittelwert; SD = Standardabweichung; Missing (%) = prozentualer Anteil fehlender Werte; 1 = „Nein und nicht geplant“, 2 = „Nein, aber konkret geplant“, 3 = „Teilweise umgesetzt“, 4 = „Vollständig oder weitgehend umgesetzt“

## Tabelle Z3. Umsetzungsgrad der Strategien zum Entlassmanagement, stratifiziert nach Struktur- und Organisationsmerkmalen

|  |  | **Prozentualer Anteil der Zustimmung**  **zur Umsetzung der Strategien** | | | | | | | | |
| --- | --- | --- | --- | --- | --- | --- | --- | --- | --- | --- |
|  |  | **Item #** | | | | | | | | |
|  | N | 1 | 2 | 3 | 4 | 5 | 6 | 7 | 8 | 9 |
| **Trägerart** |  |  |  |  |  |  |  |  |  |  |
| Öffentlich | 162 | 96 | 99 | 97 | 97 | 99 | 99 | 96 | 61 | 53 |
| Freigemeinnützig | 182 | 98 | 97 | 98 | 99 | 100 | 100 | 98 | 59 | 52 |
| Privat | 44 | 97 | 100 | 100 | 100 | 100 | 100 | 100 | 71 | 63 |
| **Bettengrößenklasse** |  |  |  |  |  |  |  |  |  |  |
| Unter 300 Betten | 159 | 97 | 99 | 99 | 98 | 99 | 100 | 96 | 60 | 52 |
| 300 bis 599 Betten | 131 | 99 | 97 | 97 | 97 | 99 | 99 | 98 | 61 | 62 |
| Ab 600 Betten | 105 | 94 | 97 | 95 | 100 | 100 | 99 | 97 | 63 | 50 |
| **Krankenhausart** |  |  |  |  |  |  |  |  |  |  |
| Universitätsklinik | 18 | 100 | 100 | 100 | 100 | 100 | 100 | 100 | 88 | 73 |
| Plankrankenhaus | 368 | 97 | 98 | 97 | 98 | 99 | 99 | 97 | 60 | 54 |
| Krankenhaus mit Versorgungsvertrag | 6 | 100 | 100 | 100 | 100 | 100 | 100 | 80 | 75 | 50 |
| **Anzahl Personen im kRM**^1,2^ |  |  |  |  |  |  |  |  |  |  |
| keine | 3 | - | - | - | - | - | - | - | - | - |
| Eine Person | 125 | 97 | 98 | 98 | 96 | 99 | 99 | 97 | 61 | 58 |
| Zwei Personen | 110 | 98 | 99 | 98 | 100 | 100 | 99 | 98 | 60 | 52 |
| Drei Personen | 53 | 98 | 98 | 98 | 100 | 100 | 100 | 100 | 74 | 64 |
| Vier Personen | 18 | 94 | 94 | 94 | 100 | 100 | 100 | 94 | 50 | 35 |
| Fünf bis 50 Personen | 41 | 92 | 95 | 92 | 97 | 97 | 100 | 95 | 54 | 57 |
| **Organisation des kRM**^1^ |  |  |  |  |  |  |  |  |  |  |
| Zentral | 240 | 95 | 97 | 97 | 97 | 99 | 99 | 96 | 57 | 55 |
| Dezentral | 15 | 100 | 100 | 100 | 100 | 100 | 100 | 100 | 75 | 55 |
| Beides | 117 | 99 | 99 | 96 | 100 | 100 | 100 | 98 | 67 | 53 |
| Durch externe Dienstleister organisiert | 1 | - | - | - | - | - | - | - | - | - |
| **Bundesland** |  |  |  |  |  |  |  |  |  |  |
| Baden-Württemberg | 30 | 96 | 100 | 96 | 100 | 100 | 100 | 96 | 54 | 58 |
| Bayern | 81 | 99 | 99 | 97 | 96 | 99 | 99 | 97 | 57 | 50 |
| Berlin | 13 | 100 | 100 | 100 | 100 | 100 | 100 | 100 | 58 | 67 |
| Brandenburg | 13 | 92 | 100 | 92 | 100 | 100 | 100 | 100 | 46 | 27 |
| Bremen | 4 | - | - | - | - | - | - | - | - | - |
| Hamburg | 9 | 100 | 100 | 80 | 100 | 100 | 100 | 100 | 60 | 60 |
| Hessen | 33 | 97 | 97 | 97 | 100 | 100 | 97 | 93 | 61 | 54 |
| Mecklenburg-Vorpommern | 2 | - | - | - | - | - | - | - | - | - |
| Niedersachsen | 38 | 100 | 100 | 97 | 100 | 100 | 100 | 100 | 76 | 61 |
| Nordrhein-Westfalen | 92 | 96 | 95 | 100 | 95 | 99 | 100 | 97 | 63 | 54 |
| Rheinland-Pfalz | 27 | 96 | 100 | 96 | 100 | 100 | 100 | 96 | 67 | 64 |
| Saarland | 5 | - | - | - | - | - | - | - | - | - |
| Sachsen | 19 | 94 | 100 | 100 | 100 | 100 | 100 | 100 | 73 | 60 |
| Sachsen-Anhalt | 10 | 90 | 89 | 89 | 100 | 100 | 100 | 90 | 33 | 33 |
| Schleswig-Holstein | 11 | 91 | 100 | 91 | 100 | 100 | 100 | 100 | 73 | 70 |
| Thüringen | 8 | 100 | 100 | 100 | 100 | 100 | 100 | 100 | 67 | 40 |

^1^ Subgruppen mit N <= 5 wurden nicht berücksichtigt. Dies betrifft: Anzahl Personen im klinischen Risikomanagement (kRM): „keine“ und Organisation des kRM: „Durch externen Dienstleister organisiert“ sowie die Bundesländer Bremen, Mecklenburg-Vorpommern und das Saarland. ^2^ Die Angaben von 5 bis 50 Personen wurden zusammengefasst.
